# Seasonality, Dietary Overlap and the Role of Taxonomic Resolution in the Study of the Diet of Three Congeneric Fishes from a Tropical Bay

**DOI:** 10.1371/journal.pone.0056107

**Published:** 2013-02-06

**Authors:** Maíra Pombo, Márcia Regina Denadai, Alexander Turra

**Affiliations:** 1 Instituto Oceanográfico da Universidade de São Paulo, São Paulo, Brazil; 2 Centro Universitário Módulo, Caraguatatuba, São Paulo, Brazil; Aristotle University of Thessaloniki, Greece

## Abstract

Not only seasonality but also taxonomic resolution of prey categories has been shown to affect diet studies. We analyzed the stomach contents of three sympatric species, *Stellifer rastrifer*, *S. brasiliensis* and *S. stellifer*, sampled monthly from August 2003 to October 2004 in Caraguatatuba Bay, southeastern Brazil. General characteristics and similarities among their diets were evaluated by considering high taxonomic ranks of all prey groups, and also the lower taxonomic ranks of the main prey groups. Dietary similarity was relatively high among species and low between seasons, and both evaluation criteria gave the equivalent results. The rare items, however, provided information about resource partition, and the species compositions of the most important groups were apparently good indicators of food availability.

## Introduction

In view of the profound effect of competition on population and community dynamics [1], the co-existence of two or more exploiters of quite similar resources is a focus of many ecological studies. This topic is especially interesting in situations of limited resources, which nearly always intensify competition, leading competitors to develop a wide range of strategies to avoid direct competition, including high levels of dietary specialization [2], [3]. Among the most common strategies to avoid direct competition are temporal or spatial segregation of foraging; feeding upon slightly different organisms, whether prey species or developmental stages; and the development of distinct morphological/physiological characteristics between very similar predator species [4]–[7].

*Stellifer* is a very diverse genus of the family Sciaenidae, which includes demersal, carnivorous, and coastal fishes, with two or more species usually sharing the same habitat [8], [9]. Its members are often very abundant in tropical coastal waters and comprise a high proportion of the discarded bycatch from seabob shrimp fisheries [10]–[12], which may compromise ecosystem control mechanisms such as “bottom-up” and “top-down” effects, to a degree that is often little understood. Nevertheless, the sciaenids' lack of commercial importance leads to fewer studies of the relationships that allow such very similar and abundant species to coexist. Most studies on the diet of *S. rastrifer*, *S. brasiliensis* and/or *S. stellifer* deal with demersal fish communities as a whole, using small samples or broad prey categories, or do not include gravimetric measurements [13]–[17]. All these reports indicated that the diets of sympatric species tend to be similar and based on crustaceans, which offered an opportunity to investigate how they share the available resources. The factors that may bias dietary studies in these ecosystems include temporal variations, which have an important role in fish feeding behavior and therefore, in affecting interactions among competitors [18] and the degree of taxonomic resolution, which may bias the observation of seasonal oscillations [19], [20].

Presuming, then, that both seasonal variations and taxonomic resolution influence studies on the diet of fishes, and also to investigate how different dietary groups, including those that are accidentally ingested, and different levels of taxonomic resolution can relate to community studies, the aim of this study was (i) to observe general characteristics and similarities of the diets of three sympatric species; to assess whether seasonality influences the species' diet and dietary similarities, considering (ii) high taxonomic ranks of all prey groups, including rare items, and (iii) lower taxonomic ranks of the main prey groups, i.e., as refined as possible, so that information provided by both evaluation criteria could be compared.

## Materials and Methods

### Sampling procedures

Sampling was performed monthly from August 2003 through October 2004, under license from the appropriate federal environmental agency (IBAMA-DIREN No. 08/2001), in Caraguatatuba Bay (23° 37′S to 23° 44′S and 45° 24′W to 45° 26′W). This bay is 16 km long, surrounded by a large urban center, and is one of the most important areas for artisanal fisheries in São Paulo state. Two homogeneous areas were selected, avoiding the influence of continental waters. Each 2 km-long area was divided into 10-m sectors, and three of these sectors were randomly selected for sampling. The boat was moved 800 m off the beach and performed eight hundred-meter otter trawls, which corresponded to a depth change of approximately 1 to 4 m. The average speed was 1 knot, and the trawl net, with two otter boards weighing 20 kg each, was 1.6 m high, 6.0 m long, 3.5 m wide, and with 2.0 cm stretched mesh size/distance between knots. The fish were first fixed in 10% formalin and, after sorting, all the specimens of *Stellifer* were labeled and fixed in 70% ethanol.

### Data analysis

Forty individuals of *S. rastrifer* and *S. brasiliensis* were selected randomly from the sample corresponding to the central period of each season, in order to avoid the influence of transitional months and to highlight seasonal shifts. *Stellifer stellifer* was much less numerous during the sampling period and for this reason all 131 individuals of this species collected, regardless of whether they were from the central month of a season, were used in the study.

Each individual was measured for total length (TL) and for digestive tract total length (DTTL), after the fish was dissected and the intestine straightened. The specieś esophagi are extremely short and the stomachs very well-defined, straight and laterally positioned. Therefore, the total length of the digestive tract essentially corresponds to the length of the intestine. The ratio between these measurements (TL/DTTL) was compared among species by one-way ANOVA, followed by the SNK test [21].

The anterior digestive tract (pharynx, esophagus and stomach) was then detached for analysis of food items. Items were separated into high taxonomic ranks under a stereomicroscope, and each category in a single stomach was weighed (10 µg accuracy). Prey individuals were then identified to the most refined taxonomic level possible, and counted, for each stomach. Total weight of the food consumed by each individual was compared among species by one-way ANOVA, followed by the SNK test.

To assess differences in diet by using broader taxonomic resolution, for each prey category the frequency of occurrence (FO) was calculated as the percentage of stomachs containing food, and the weight percentage (W%) was calculated by dividing the weight of each food category by the weight of all items in the respective digestive tract [22]. The percent weight was used to estimate the trophic level of each species, with the qualitative routine of TrophLab [23]. Both FO and W% were plotted on graphs [24] and used to calculate the index of dietary importance for each food category (IAi; [25]): IAi = FOi*Wi/∑FOi*Wi. This index was in turn used to calculate the similarity index (PS) both among seasons and among species [26], [27]. Items such as “fragments” or “organic matter” were assigned to the most refined category to which they could belong before the PS was calculated, preventing differences in food digestibility from masking the results for dietary overlap.

To assess differences in diet by using a more refined taxonomic resolution of the main groups ingested, each prey species, or subgroup, was evaluated for FO and numerical percentage within the higher group to which it belonged (N%). These parameters were then used, again, to prepare graphs, to calculate a specific dietary importance index (IAis) and to compare similarity among diets (PS), again for seasons and species. Shannon indices of diversity and equitability were also calculated for seasons and species, to provide information independent of comparisons between predator or season.

## Results

The range, mean and standard deviation of total lengths were similar among *S. rastrifer* (5.00 to 14.05; 8.23 ± 2.07), *S. brasiliensis* (4.90 to 12.00; 7.91 ± 1.68 cm) and *S. stellifer* (3.85 to 12.6; 8.21 ± 1.68 cm). The TL/TDTL ratio was significantly different among the species (F = 93.52, d.f. = 2, n = 431, p<0.001), and was highest for *S. rastrifer* (0.48 ± 0.01 cm, n = 160), intermediate for *S. brasiliensis* (0.42 ± 0.01 cm, n = 159), and lowest for *S. stellifer* (0.39 ± 0.01 cm, n = 115; SNK, p<0.001).

The frequency of empty stomachs was 5.63% for *S. rastrifer*, which showed only 9 empty stomachs in all. Empty stomachs were much more frequent in both of the other species, reaching 28.7% for *S. stellifer* and 40% for *S. brasiliensis* (33 and 64 empty stomachs respectively). Food weight per stomach also differed significantly among the species (H = 34.55, d.f. = 2, n = 245, p<0.001), reaching higher values for *S. rastrifer* (0.018 ± 0.066 g, n = 117; SNK, p<0.001 for both comparisons) and equal and lower values for the other two species, *S. brasiliensis* (0.006 ± 0.019 g, n = 68) and *S. stellifer* (0.006 ± 0.020 g, n = 60; SNK, p = 0.39).

### Analysis using high taxonomic ranks

The vast majority of food items were Crustacea; Mysida, Copepoda and Decapoda were the most important taxa in the diet of all three species (Table S1). *S. rastrifer* also showed a high frequency (over 20%) of ingestion of Amphipoda and Chaetognatha, but both these groups contributed little to the percentage weight. Other crustacean taxa were Isopoda, Cumacea, Ostracoda, as well as a few tiny ascidians, semi-digested nematodes and Osteichthyes, mostly represented by scales, unimportant in weight and possibly present due to sampling artifacts. Trophic level estimated for *S. rastrifer* was 3.09 (±0.28SE). For *S. brasiliensis*, fewer rare taxa were identified: Amphipoda, Osteichthyes, Polychaeta, Chaetognatha and bivalve siphons. *S. brasiliensis* showed the largest amount of highly digested items. Its trophic level was 3.10 (±0.30SE). *S. stellifer* showed basically the same items as the other two species, differing in having the highest frequency of ascidians (17%), fewer Amphipoda and more Chaetognatha, reaching a trophic level of 3.06 (±0.31SE). Thus, the overall values of the similarity index (PS) were high, reaching 0.82 between *S. rastrifer* and *S. stellifer*. For *S. rastrifer* and *S. brasiliensis* the PS was 0.74, with the lowest value (0.66) between *S. brasiliensis* and *S. stellifer*. When calculated per season, most of these PS values between species were even higher than the overall value, especially in the summer (Table 1). Comparisons including *S. stellifer* resulted in the lowest observed values.

**Table 1:** Similarity indexes (PS) between pairs of congeneric species, *Stellifer rastrifer*, *S. brasiliensis* and *S. stellifer* (Sciaenidae, Perciformes) collected in Caraguatatuba Bay from August 2003 through October 2004.

Season*	Species		
	*S. rastrifer x*	*S. rastrifer x*	*S. brasiliensis x*
	*S. brasiliensis*	*S. stellifer*	*S. stellifer*
Sp	0.94	0.43	0.38
Su	0.94	0.84	0.80
Au	0.74	0.50	0.72
Wi	0.83	0.32	0.19
Ov	0.74	0.82	0.66

* - Sp – Spring; Su – Summer, Au – Autumn, Wi – Winter, Ov – Overall.

**Figure 1:**
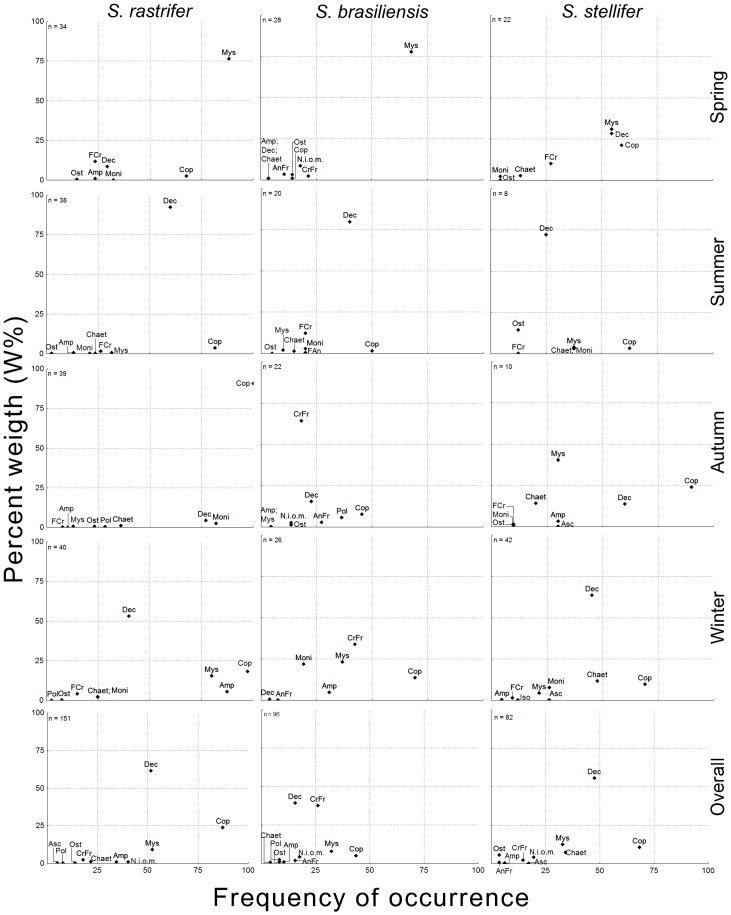
Higher taxonomic ranks of prey. Weight percentage (W%) and frequency of occurrence (FO) of higher taxonomic ranks of prey. Composition is shown by season and in general for *Stellifer rastrifer*, *S. brasiliensis* and *S. stellifer* (Sciaenidae, Perciformes), collected in Caraguatatuba Bay from August 2003 to October 2004. **Abbrev.**: Mysida (Mys), Decapoda (Dec), Copepoda (Cop), Amphipoda (Amp), Isopoda (Iso), Chaetognatha (Chaet), Ascidiacea (Asc), Polychaeta (Pol), Osteichthyes (Ost), unidentified crustacean fragment, (CrFr), unidentified animal fragment (AnFr), unidentified organic matter (N.i.o.m.).

The importance of each of the most numerous items alternated among seasons, following a very similar pattern among species (Figure 1): Mysida was the main item in spring, less abundant in winter, and virtually absent in autumn and summer. Decapoda was most important in summer, due to the presence of a large number of sergestoids, since other decapod groups were much less numerous. Therefore, although decapods showed a high frequency of occurrence, percentage weight was the main factor that distinguished this group in this season. Copepods predominated only in autumn, although they were highly frequent during the entire period. Finally, in winter these predominant groups were more equally distributed in the diets of all three species, and included an increase of amphipods in the diets of *S. rastrifer* and *S. brasiliensis* and chaetognaths in the diet of *S. stellifer*. These observations were confirmed by the PS calculations for inter-season similarity, which was low for all the species, except for *S. stellifer* in some of the comparisons (Table 2).

**Table 2:** Matrix of similarity index (PS) between seasons obtained from the higher taxonomic ranks that constituted the diets of *Stellifer rastrifer*, *S. brasiliensis* and *S. stellifer* (Sciaenidae, Perciformes), collected in Caraguatatuba Bay from August 2003 through October 2004.

Season*	Species								
	*S. rastrifer*			*S. brasiliensis*			*S. stellifer*		
	Sp	Su	Au	Sp	Su	Au	Sp	Su	Au
Su	0.07			0.02			0.49		
Au	0.05	0.14		0.01	0.23		0.73	0.39	
Wi	0.37	0.25	0.47	0.43	0.06	0.51	0.53	0.91	0.43

* - Sp – Spring; Su – Summer, Au – Autumn, Wi – Winter.

### Analysis using low taxonomic ranks

Concerning the specific composition of the predominant items, all the identifiable mysids were *Mysidopsis coelhoi.* The amphipods were predominantly *Tiron* spp. and some *Cerapus* spp. Other amphipod species were identified only in the diet of *S. rastrifer*, and only in a few isolated cases (Table S2). Therefore, Decapoda and Copepoda, the most important groups and with the largest numbers of identified species/subgroups, were considered the most reliable groups to assess the diets of these fishes by means of species composition.

**Figure 2:**
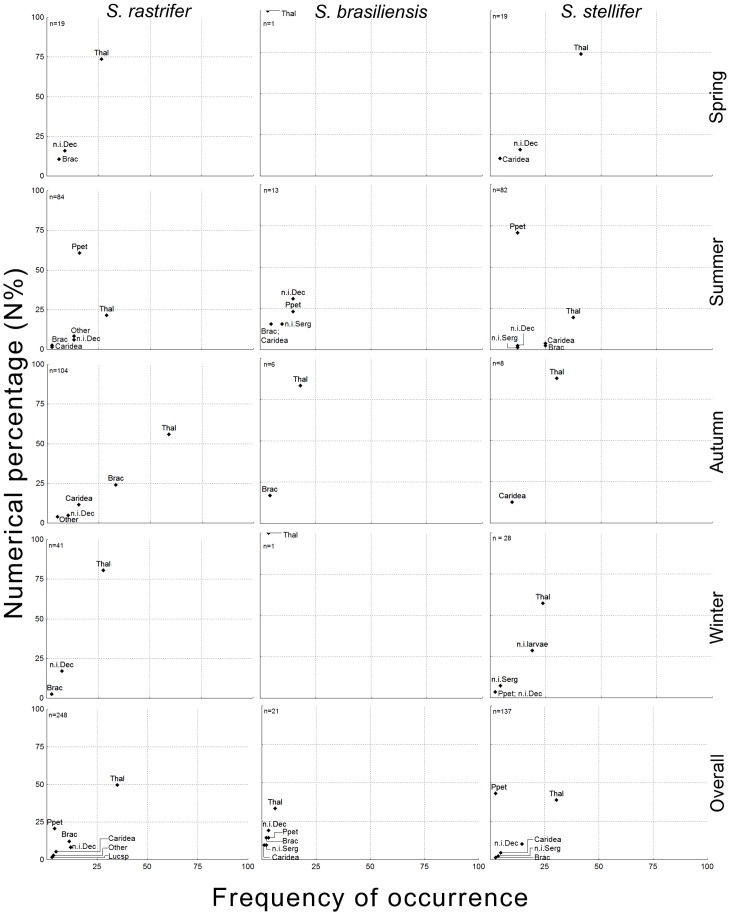
Lower taxonomic ranks of Decapoda prey. Numerical percentage (N%) and frequency of occurrence (FO) of lower taxonomic ranks of Decapoda. Composition is shown by season and in general for *Stellifer rastrifer*, *S. brasiliensis* and *S. stellifer* (Sciaenidae, Perciformes), collected in Caraguatatuba Bay from August 2003 to October 2004. **Abbrev.**: Thalassinidae (Thal), *Peisos petrunkevichi* (Ppet), Brachyura (Brac), unidentified Caridea (Caridea), *Lucifer* sp. (Lucsp), unidentified Sergestoidea (n.i. Serg), unidentified Decapoda (n.i. Dec), unidentified Decapoda larvae (n.i. larvae).

Most decapods observed were early-stage juveniles, difficult to identify to species level. The main subgroup was Thalassinidae, with a high frequency of occurrence for all three predators and the highest numerical percentage of the Decapoda, except in the diet of *S. stellifer*, where Sergestoidea comprised the majority. The sergestoids were all adults of *Peisos petrunkevichi*. Brachyura and Caridea were observed in smaller numbers, and other decapod subgroups were found rarely (Figure 2).

**Figure 3:**
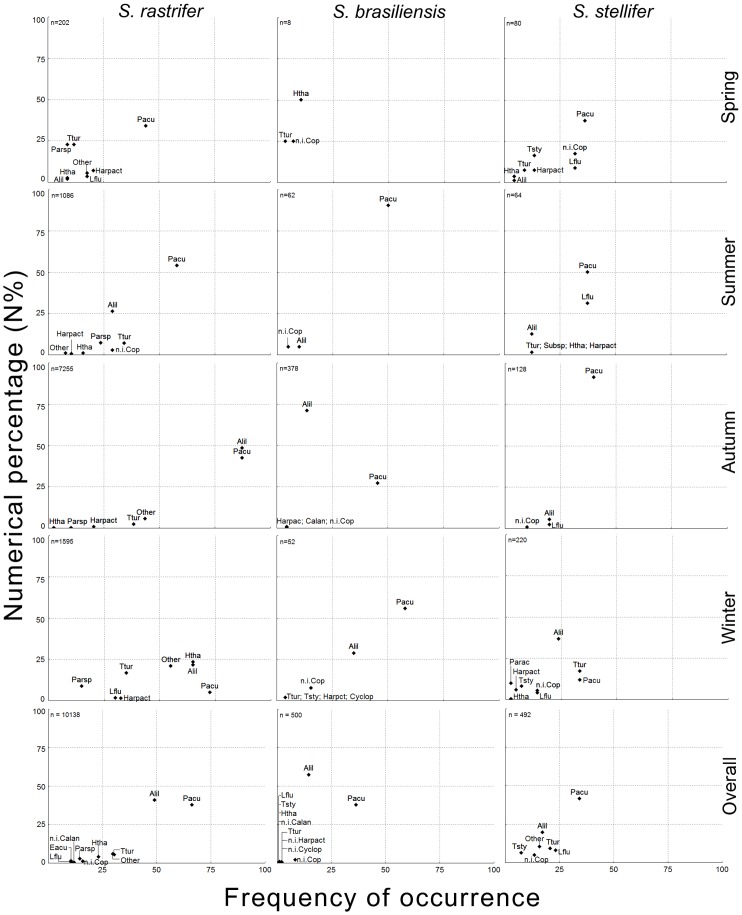
Lower taxonomic ranks of Copepoda prey. Numerical percentage (N%) and frequency of occurrence (FO) of lower taxonomic ranks of Copepoda. Composition is shown by season and in general for *Stellifer rastrifer*, *S. brasiliensis* and *S. stellifer* (Sciaenidae, Perciformes), collected in Caraguatatuba Bay from August 2003 to October 2004. **Abbrev.**: *Acartia lilljeborgii* (Alil), *Labidocera fuviatilis* (Lflu), *Paracalanus* sp. (Parsp), *Pseudodiaptomus acutus* (Pacu), *Temora turbinata* (Ttur), *Hemicyclops thalassus* (Htha), unidentified Harpacticoida (Harpact).

In the diet of *S. rastrifer*, Copepoda was represented by 10,138 individuals from at least 17 species (Table S2); 93.19% were from the order Calanoida. Two calanoids, *Acartia lilljeborgii* and *Pseudodiaptomus acutus*, were particularly important. The former was prominent mainly for its percentage composition among copepods, and the latter mainly for its frequency of occurrence (Figure 3). Other calanoids also occurred frequently, including *Temora turbinata*, *Paracalanus* spp. and *Labidocera fluviatilis*. Harpacticoids (mostly *Euterpina acutifrons*) and cyclopoids (mostly *Hemicyclops thalassus*) comprised a small proportion of all copepods. *Stellifer brasiliensis* and *S. stellifer* showed a much lower absolute number of copepods than *S. rastrifer*, but still the copepod species composition was similar for all the predators (Table S2). Therefore, the PS values between *Stellifer* species were again higher when they were calculated based on low-level taxa, ranging from 0.66 to 0.90 when calculated based on the decapod composition and 0.79 to 0.89 when calculated based on the copepod composition. Seasonally, these values increased when based on decapod species, and decreased when based on copepod species (Table 3).

**Table 3:** Similarity index (PS) between pairs of congeneric species, *Stellifer rastrifer*, *S. brasiliensis* and *S. stellifer* (Sciaenidae, Perciformes) obtained from the Decapoda and Copepoda species/subgroups constituting their diets.

Group	Season^*^	Species		
		*S. rastrifer x* *S. brasiliensis*	*S. rastrifer x* *S. stellifer*	*S. brasiliensis x* *S. stellifer*
Decapoda	Sp	0.93	0.95	0.95
	Su	0.33	0.93	0.49
	Au	0.81	0.80	0.95
	Wi	0.94	0.73	0.70
	Ov	0.69	0.90	0.66
Copepoda	Sp	0.12	0.64	0.18
	Su	0.72	0.62	0.58
	Au	0.89	0.49	0.59
	Wi	0.32	0.57	0.45
	Ov	0.89	0.70	0.77

The fish were collected in Caraguatatuba Bay from August 2003 through October 2004. * - Sp – Spring; Su – Summer, Au – Autumn, Wi – Winter, Ov – Overall.

Seasonal analysis of decapod individuals showed that Thalassinidae was the predominant subgroup throughout the year, in both FO and N%, except for summer, when the Sergestoida raised the importance of decapods for all three species. Summer and autumn were the richest seasons of Decapoda for all predators, and spring the lowest (Figure 2). Accordingly, the PS for seasonality was always less than 0.60 for the summer, but ranged from 0.70 to 1.00 between any other seasons, for all predators (Table 4).

**Table 4:** Matrix of similarity indexes (PS) between seasons obtained for Decapoda and Copepoda species/subgroups that constituted the diets of *Stellifer rastrifer*, *S. brasiliensis* and *S. stellifer* (Sciaenidae, Perciformes), collected in Caraguatatuba Bay from August 2003 through October 2004.

		Species/Season								
		*S. rastrifer*			*S. brasiliensis*			*S. stellifer*		
Group	Season*	Sp	Su	Au	Sp	Su	Au	Sp	Su	Au
Decapoda	Su	0.39			0.00			0.43		
	Au	0.80	0.39		0.95	0.05		0.96	0.45	
	Wi	0.97	0.40	0.77	1.00	0.00	0.95	0.73	0.43	0.70
Copepoda	Su	0.80			0.00			0.70		
	Au	0.48	0.64		0.00	0.85		0.58	0.61	
	Wi	0.28	0.36	0.61	0.03	0.75	0.79	0.32	0.27	0.23

* - Sp – Spring; Su – Summer, Au – Autumn, Wi – Winter.

Regarding copepods, autumn was by far the season with the highest number of individuals. Spring had the lowest, and summer and winter had similar, intermediate numbers of individuals. When the data for *S. stellifer* were standardized to 40 stomachs per season, to compensate for the unequal numbers of stomachs examined in the different seasons, the same seasonal pattern of copepod abundance was seen as in the other species.

*S. rastrifer* showed the highest absolute number and richness of copepods. Interestingly, from the spring onward, *A. lilljeborgii* increased in importance, reaching a maximum in autumn, in parallel with a decrease in the variety and importance of other species. In winter this situation reversed and more closely resembled the spring pattern. *Pseudodiaptomus acutus* was more constantly important during the year than was *A. lilljeborgii*, although *P. acutus* increased from spring to autumn and decreased in winter. Inversely, Cyclopoida, Harpacticoida and some other calanoid species such as *Paracalanus* spp. were more abundant in spring and winter. This pattern of copepod species distribution through seasons was, again, similar for the other two predators (Figure 3).

The PS values for seasonality assessed from copepods were not as homogeneous among the three predators as were those for Decapoda. However, for all of them, values were lower than 0.60 between spring and winter/fall, and higher between summer and fall (Table 4).

The Shannon diversity index was highest for *S. rastrifer* and lowest for *S. brasiliensis*. Interestingly, higher and lower diversity values by seasons alternated between Decapoda and Copepoda (Table 5), with Decapoda showing higher diversity in summer and autumn and Copepoda in spring and winter. Shannon equitability was much higher for *S. stellifer* than for the other two species, for both Decapoda and Copepoda. The inverse was observed for *S. brasiliensis*.

**Table 5:** Shannon diversity (H) and equitability (EH) indexes of Copepoda and Decapoda constituting the diet of *Stellifer rastrifer*, *S. brasiliensis* and *S. stellifer* (Sciaenidae, Perciformes) collected in Caraguatatuba Bay from August 2003 through October 2004.

Group	Season*	Index/Species					
		Diversity (H)			Equitability (EH)		
		*S. rastrifer*	*S. brasiliensis*	*S. stellifer*	*S. rastrifer*	*S. brasiliensis*	*S. stellifer*
Decapoda	Sp	0.38	0.00	0.38	0.54	-	0.54
	Su	1.03	0.99	0.82	0.56	0.90	0.51
	Au	1.09	0.45	0.38	0.60	0.65	0.54
	Wi	0.13	0.00	0.54	0.19	-	0.49
	Ov	1.30	0.67	1.02	0.59	0.48	1.47
Copepoda	Sp	1.73	0.64	1.56	0.67	0.91	0.80
	Su	1.18	0.20	1.23	0.54	0.29	0.63
	Au	0.99	0.61	0.32	0.42	0.55	0.29
	Wi	1.94	0.91	1.76	0.73	0.56	0.80
	Ov	1.40	0.78	1.67	0.49	0.44	0.73

* - Sp – Spring; Su – Summer, Au – Autumn, Wi – Winter, Ov – Overall.

## Discussion

The amount of ingested food and the degree of digestion of the food items differed widely among the three species. Since the three congenerics showed very similar size ranges and means, and it is also known that they behave very similarly concerning population biology in the study area [28], these results do not reflect ontogenetic shifts. These differences could be due to different periods of foraging activity, a common strategy for resource partitioning [2], [5]. However, as the DTTL/TL ratios were also different, these differences apparently result not only from behavioral, but also morphological differences, even though all species showed a ratio indicating a fully carnivorous diet, i.e., <1 [29]. The similarity among individuals in size and dietary composition suggests that these morphological differences are phylogenetic and influence the diversity of dietary strategies of the different predators. For related species of Stichaeidae, phylogeny constrains the digestive features more strongly than does phenotypic plasticity [7]. These structural differences are very likely to be accompanied by some physiological adaptation. For two species of *Cyprinus*, a physiological difference confers an advantage on one species, which feeds less often but converts food into energy more efficiently [6].

With respect to the items ingested, other studies have reported that crustaceans are an important component of the diet of species of *Stellifer* [13], [15]–[17], but a particularity observed here was that other commonly consumed items such as Polychaeta, Mollusca and Teleostei were virtually absent. Since greater or lesser food availability may affect dietary composition [30], the very pronounced consumption of Crustacea in this study may indicate favorable feeding conditions. Notably, although penaeoids are an important bycatch component of the shrimp fishery (*Xiphopenaeus kroyeri*), only one penaeoid individual was found in the stomachs of these fish. Other investigators have attributed the absence of penaeoids to their closer association with the substrate and to the sharper rostrum [15]. Therefore, *S. rastrifer* probably plays an important role in protecting the penaeoid population, as suggested by Coelho [31], because it may successfully compete with potential predators and prey upon competitor shrimps.

### Analysis using high taxonomic ranks

The high similarity index among these species of *Stellifer*, which was also reflected in the similarity among the estimations of trophic levels, contrasts with the findings of Micheletti & Uieda [14]. The use of poorly specified categories such as “organic matter” or “Crustacea fragments” may seriously bias analyses. Here, apportioning of these items into more specific categories was essential, because the much further advanced state of digestion of the stomach contents found in *S. brasiliensis* does not necessarily mean that it is not feeding on the same items, as the calculation of indices would imply. With such a high similarity, it is unlikely that effective competition is occurring among the species. On the contrary, this may again reflect favorable environmental conditions of food availability.

On the other hand, season-to-season similarity was very low for all three species. Similarity was higher when the comparisons included winter, when the items were distributed more homogeneously. Prey availability and size both affect the ingestion of an item, since it is more profitable to catch small numbers of large prey instead of a large number of small prey [18]. Therefore, the results may reflect wide variations of prey species during the year, although no data on plankton are available for the area. Based on these results, a rough estimate would predict shifts from 3.0 (hypothetical consumption of only copepods) to slightly over 3.2 (value for a hypothetical ingestion of Mysida exclusively) during the year.

Calculation of similarity between species by season was very important to reveal the lower overall values. The overall IAi values were strongly affected by the wide seasonal differences in the ingested items, due to the overvaluation of larger items by gravimetric methods and of the smaller items by numerical methods. Notably for Mysida, the intermediate size and, therefore, intermediate numbers resulted in underestimation of its overall index value when compared with Decapoda or Copepoda, when actually mysids were ingested in larger or smaller amounts in different seasons. Also, these seasonal values revealed that the highest similarity occurred between *S. rastrifer* and *S. brasiliensis*, rather than between *S. rastrifer* and *S. stellifer* as predicted by the overall values. Again, lower values were observed for comparisons involving winter (greater homogeneity) and *S. stellifer* (not all individuals were from the central month of each season).

The composition of rare items elucidated how the predators share foraging space. *S. brasiliensis* ingested the highest amount of Annelida and the lowest amount of Chaetognatha, and also was the only species that ingested siphons, indicating that it probably feeds closest to the bottom; this habit concords with its mouth position, the most ventrally located [32]. Near-bottom feeding may be also related to the more degraded condition of the items. *S. stellifer* ingested the highest amount of Chaetognatha, the lowest amount of Amphipoda, and did not ingest tube-building amphipods or cladocerans, which is probably related to a habit of foraging closer to the surface, coinciding with its more oblique mouth position. *S. rastrifer*, with an intermediate mouth position, showed intermediate similarity to the other species. Even though the more important items resulted in fairly high values of similarity among the three species, these small behavioral or morphological differences may be essential in decreasing competition among predators during occasional conditions of food scarcity [33].

### Analysis using low taxonomic ranks

The species composition of Copepoda and Decapoda generated even higher values of similarities between species, but the composition of these groups was affected by seasonality in different ways. For Decapoda, the dissimilarity was due to the almost exclusive occurrence of *Peisos petrunckevichi* in summer and the predominance of Thalassinidae in other seasons. For *S. brasiliensis* this similarity was probably underestimated because of the poor condition of the items in this season, mostly classified as Sergestoidea not identified (n.i.). Copepoda showed more subtle, but constant differences in composition over the seasons, partly as a consequence of the gradual increases or decreases of the most abundant copepod species, *A. lilljeborgii* and *P. acutus*. The homogeneity or heterogeneity of each group over the seasons was reflected in the values of similarity between the predators. Species richness also agreed with these results, since more species of copepods than decapods could be identified.

In general, the values of similarities between species derived from the lower taxa confirmed the results derived from the higher taxa, although the patterns were less clear. The diversity index was useful to confirm these interpretations, because periods of greater diversity undoubtedly led to lower similarity values when these were based on lower taxa.

The diversity index followed the same temporal patterns for all *Stellifer* species, which proved to be generalists. Seasonality in diet is related to food availability [18], and indeed, the present results are in accordance with the expected environmental conditions in a human-impacted tropical bay. The occurrence of few species of most taxa, along with their constancy (Mysida, Amphipoda, Decapoda), is a common condition in disturbed areas such as this one [34]–[37]. Also, the number and composition of copepod species was similar to studies of plankton, that used conventional sampling methods, in similar areas [38]–[40]. Thus, Copepoda seems to be the group that best corresponds to the true proportion occurring in the bay. Their homogeneity in size implies that copepods were more randomly ingested, and consequently seasonal variations followed those expected for the copepod community in similar areas: an increasing diversity in the drier seasons, including the presence of some species associated with colder, more oceanic waters (*Temora stylifera, Ctenocalanus* spp.), and few, very numerous species in wetter seasons, such as *A. lilljeborgii* and *P. acutus* [41]. The alternation in abundance over time between the genera *Paracalanus* and *Acartia* has also been reported by other investigators [38], [42], as has the constancy in the occurrence of *E. acutifrons* in low densities over time [39].

The species composition of Decapoda and Copepoda showed an interesting inverse pattern of diversity among seasons, for all three *Stellifer* species. A reasonable explanation is that many decapod species have a preference for low-salinity waters, either for living or for reproduction, and these waters would extend farther into coastal waters during rainy seasons [43]–[45]. Conversely, the copepod assemblage included more species with a greater affinity with more-oceanic conditions, and would move closer to continental areas during drier periods [41].

## Supporting Information

Table S1 **Higher taxonomic ranks of items identified in the diets of the three species** ***Stellifer rastrifer*** **(size range: 5.0 to 14.0 cm),** ***S. brasiliensis*** **(4.9 to 12.0 cm) and** ***S. stellifer*** **(3.8 to 12.6 cm) (Sciaenidae, Perciformes) collected in Caraguatatuba Bay from August 2003 through October 2004, and the respective overall values of: number of observations (n), frequency of occurrence (FO), weight percentage (W%) and index of dietary importance (IAi) of each prey group.** Name of predator species are followed, in brackets, by number of fishes examined and FO by number of non empty stomachs. * - Items assessed by presence or absence, i.e., number of observations (n) is equivalent to the number of stomachs in which they occurred.
(DOC)

Table S2 **Lower taxonomic ranks of the most important groups found in the stomach contents of** ***Stellifer rastrifer*** **,** ***S. brasiliensis*** **and** ***S. stellifer*** **(Sciaenidae, Perciformes), collected in Caraguatatuba Bay from August 2003 through October 2004, and the respective overall values of: frequency of occurrence (FO), numerical percentage in the respective group (N%) and specific index of dietary importance (IAis) of each prey group.**
(DOC)
