# Supplementary material for: Seasonality, Dietary Overlap and the Role of Taxonomic Resolution in the Study of the Diet of Three Congeneric Fishes from a Tropical Bay
Source: PLoS One. 2013 Feb 6;8(2):e56107. doi: 10.1371/journal.pone.0056107 (PMC3566041; doi:10.1371/journal.pone.0056107)
Supplement: Table S1 — Higher taxonomic ranks of items identified in the diets of the three species Stellifer rastrifer (size range: 5.0 to 14.0 cm), S. brasiliensis (4.9 to 12.0 cm) and S. stellifer (3.8 to 12.6 cm) (Sciaenidae, Perciformes) collected in Caraguatatuba Bay from August 2003 through October 2004, and the respective overall values of: number of observations (n), frequency of occurrence (FO), weight percentage (W%) and index of dietary importance (IAi) of each prey group. Name of predator species are followed, in brackets, by number of fishes examined and FO by number of non empty stomachs. * - Items assessed by presence or absence, i.e., number of observations (n) is equivalent to the number of stomachs in which they occurred. (DOC) [file pone.0056107.s001.doc]

Table S1. Higher taxonomic ranks of items identified in the diets of the three species *Stellifer rastrifer* (size range: 5.0 to 14.0 cm), *S. brasiliensis* (4.9 to 12.0 cm) and *S. stellifer* (3.8 to 12.6 cm) (Sciaenidae, Perciformes) collected in Caraguatatuba Bay from August 2003 through October 2004, and the respective overall values of: number of observations (n), frequency of occurrence (FO), weight percentage (W%) and index of dietary importance (IAi) of each prey group. Name of predator species are followed, in brackets, by number of fishes examined and FO by number of non empty stomachs. * - Items assessed by presence or absence, i.e., number of observations (n) is equivalent to the number of stomachs in which they occurred.

| Item group | Species | | | | | | | | | | | |
| --- | --- | --- | --- | --- | --- | --- | --- | --- | --- | --- | --- | --- |
|  | *S. rastrifer* (160) | | | | *S. brasiliensis* (160) | | | | *S. stellifer* (131) | | | |
|  | n | FO (151) | W% | IAi | n | FO (96) | W% | IAi | n | FO (98) | W% | IAi |
| Copepoda | 10138 | 87.42 | 23.68 | 0.35 | 500 | 43.75 | 5.03 | 0.12 | 493 | 68.29 | 10.67 | 0.17 |
| Decapoda | 248 | 51.66 | 61.51 | 0.54 | 21 | 15.62 | 39.26 | 0.15 | 137 | 47.56 | 55.54 | 0.63 |
| Amphipoda | 308 | 34.44 | 0.95 | 0.00 | 16 | 10.41 | 0.75 | 0.00 | 13 | 6.10 | 0.49 | 0.00 |
| Mysida | 386 | 52.32 | 9.17 | 0.08 | 18 | 32.29 | 7.68 | 0.13 | 129 | 32.93 | 12.53 | 0.10 |
| Isopoda | 20 | 9.27 | 0.01 | 0.00 | 3 | 2.08 | 0.03 | 0.00 | 9 | 6.10 | 0.07 | 0.00 |
| Cumacea | 12 | 3.97 | 0.00 | 0.00 | 4 | 3.12 | 0.09 | 0 | - | - | - | - |
| Ostracoda | 1 | 0.66 | 0.00 | 0.00 | - | - | - | - | - | - | - | - |
| Tanaidacea | - | - | - | - | - | - | - | - | 1 | 1.22 | 0.11 | 0.00 |
| Crustacea fragments^*^ | 27 | 17.88 | 2.36 | 0.01 | 25 | 26.04 | 37.47 | 0.53 | 12 | 14.63 | 2.32 | 0.01 |
| Polychaeta | 15 | 7.95 | 0.08 | 0.00 | 9 | 8.33 | 2.30 | 0.01 | - | - | - | - |
| Ascidian | 11 | 5.19 | 0.05 | 0.00 | - | - | - | - | 16 | 17.07 | 0.08 | 0.00 |
| Chaetognatha | 173 | 21.85 | 1.06 | 0.00 | 12 | 4.17 | 0.60 | 0.00 | 157 | 34.15 | 7.27 | 0.06 |
| Nematoda | 3 | 2.65 | 0.00 | 0.00 | - | - | - | - | - | - | - | - |
| Osteichthyes (scales, larvae, fragments) | 29 | 13.91 | 0.22 | 0.00 | 8 | 8.33 | 0.84 | 0.00 | 7 | 3.66 | 5.65 | 0.00 |
| Bivalvia siphon | - | - | - | - | 2 | 2.08 | 0.13 | 0.00 | - | - | - | - |
| Shell fragments^*^ | 1 | 0.66 | 0.00 | 0.00 | - | - | - | - | - | - | - | - |
| Animal fragments^*^ | 1 | 0.66 | 0.04 | 0.00 | 6 | 15.62 | 1.71 | 0.01 | 3 | 3.66 | 0.90 | 0.00 |
| Organic matter^*^ | 61 | 40.40 | 0.87 | 0.01 | 17 | 17.70 | 4.09 | 0.04 | 16 | 19.51 | 4.30 | 0.02  dlgjbneljdgtbnredbrjntb  xvxbdxbdgbjdnbjldnbvkdljnbd |
