# Supplementary material for: Seasonality, Dietary Overlap and the Role of Taxonomic Resolution in the Study of the Diet of Three Congeneric Fishes from a Tropical Bay
Source: PLoS One. 2013 Feb 6;8(2):e56107. doi: 10.1371/journal.pone.0056107 (PMC3566041; doi:10.1371/journal.pone.0056107)
Supplement: Table S2 — Lower taxonomic ranks of the most important groups found in the stomach contents of Stellifer rastrifer , S. brasiliensis and S. stellifer (Sciaenidae, Perciformes), collected in Caraguatatuba Bay from August 2003 through October 2004, and the respective overall values of: frequency of occurrence (FO), numerical percentage in the respective group (N%) and specific index of dietary importance (IAis) of each prey group. (DOC) [file pone.0056107.s002.doc]

Table S2. Lower taxonomic ranks of the most important groups found in the stomach contents of *Stellifer rastrifer*, *S. brasiliensis* and *S. stellifer* (Sciaenidae, Perciformes), collected in Caraguatatuba Bay from August 2003 through October 2004, and the respective overall values of: frequency of occurrence (FO), numerical percentage in the respective group (N%) and specific index of dietary importance (IAis) of each prey group

| Item category | Species | | | | | | | | |
| --- | --- | --- | --- | --- | --- | --- | --- | --- | --- |
|  | *S. rastrifer* | | | *S. brasiliensis* | | | *S. stellifer* | | |
| Amphipoda | FO | N% | IAis | FO | N% | IAis | FO | N% | IAis |
| *Cerapus spp.* | 3.97 | 7.47 | 0.01 | 1.04 | 6.25 | 0.01 | - | - | - |
| *Ericthonius sp.* | 0.66 | 0.32 | 0.00 | - | - | - | - | - | - |
| *Photis brevipes* | 0.66 | 0.32 | 0.00 | - | - | - | - | - | - |
| *Tiron spp.* | 29.80 | 87.01 | 0.98 | 6.25 | 68.75 | 0.84 | 4.88 | 76.92 | 0.82 |
| Melitidae (n.i.) | 3.97 | 2.27 | 0.00 | - | - | - | - | - | - |
| Corophiidae (n.i.) | 0.66 | 0.32 | 0.00 | - | - | - | - | - | - |
| Gammaridea (n.i.) | 0.66 | 0.32 | 0.00 | - | - | - | - | - | - |
| Amphipoda (n.i.) | 3.97 | 1.95 | 0.00 | 3.12 | 25.00 | 0.15 | 3.66 | 23.08 | 0.18 |
| Decapoda |  |  |  |  |  |  |  |  |  |
| *Lucifer spp.* | 2.65 | 1.61 | 0.00 | - | - | - | - | - | - |
| *Peisos petrunkevitchi* | 3.97 | 20.56 | 0.04 | 3.12 | 14.29 | 0.12 | 2.44 | 43.07 | 0.07 |
| Sergestidae (n.i.) | 0.66 | 0.40 | 0.00 | 2.08 | 9.52 | 0.05 | 3.66 | 2.19 | 0.01 |
| Penaeoidea (n.i.) | 0.66 | 0.40 | 0.00 | - | - | - | - | - | - |
| Dendrobranchiata (n.i.) | 0.66 | 0.81 | 0.00 | - | - | - | - | - | - |
| Caridea (n.i.) | 4.64 | 5.24 | 0.01 | 1.04 | 9.52 | 0.03 | 4.88 | 4.38 | 0.01 |
| (Larvae) Thalassinidae (n.i.) | 35.10 | 49.60 | 0.85 | 6.25 | 33.33 | 0.56 | 30.49 | 38.69 | 0.82 |
| (Larvae) Brachyura (n.i.) | 11.26 | 12.10 | 0.07 | 2.08 | 14.29 | 0.08 | 2.44 | 1.46 | 0.00 |
| (Larvae) Porcellanidae (n.i.) | 0.66 | 0.40 | 0.00 | - | - | - | - | - | - |
| (Larvae) Paguroidea (n.i.) | 0.66 | 0.81 | 0.00 | - | - | - | - | - | - |
| Decapoda larvae (n.i.) | 7.28 | 5.24 | 0.02 | - | - | - | 13.42 | 8.76 | 0.08 |
| Decapoda (n.i.) | 4.64 | 2.82 | 0.01 | 3.12 | 19.05 | 0.16 | 2.44 | 1.46 | 0.00 |
| Copepoda |  |  |  |  |  |  |  |  |  |
| Calanoida | 80.13 | 93.19 | 0.98 | 39.58 | 96.60 | 1.00 | 51.22 | 90.06 | 0.99 |
| *Acartia lilljeborgii* | 49.01 | 40.96 | 0.41 | 14.58 | 57.40 | 0.37 | 17.07 | 19.68 | 0.15 |
| *Acartia tonsa* | 4.64 | 2.77 | 0.00 | - | - | - | - | - | - |
| *Acartia spp.* | 0.66 | 0.33 | 0.00 | - | - | - | - | - | - |
| *Labidocera fluviatilis* | 11.92 | 0.35 | 0.00 | - | - | - | 23.17 | 8.11 | 0.08 |
| *Ctenocalanus spp.* | 2.65 | 0.31 | 0.00 | - | - | - | - | - | - |
| *Paracalanus spp.* | 14.57 | 2.69 | 0.01 | - | - | - | - | - | - |
| *Parvocalanus crassirostris* | 3.97 | 0.76 | 0.00 | - | - | - | - | - | - |
| *Pseudodiaptomus acutus* | 66.23 | 37.70 | 0.51 | 36.46 | 37.80 | 0.62 | 34.15 | 41.58 | 0.63 |
| *Pseudodiaptomus spp.* | 0.66 | 0.01 | 0.00 | - | - | - | - | - | - |
| *Temora turbinata* | 30.46 | 5.49 | 0.03 | 2.08 | 0.60 | 0.00 | 20.73 | 9.13 | 0.08 |
| *Temora stylifera* | 3.31 | 0.13 | 0.00 | 1.04 | 0.20 | 0.00 | 7.32 | 6.29 | 0.02 |
| *Subeucalanus sp.* | - | - | - | - | - | - | 1.22 | 0.2 | 0.00 |
| Paracalanidae (n.i.) | 1.32 | 0.40 | 0.00 | - | - | - | 1.22 | 4.46 | 0.00 |
| Pontellidae (n.i.) | 1.99 | 0.01 | 0.00 | - | - | - | - | - | - |
| Calanoida (n.i.) | 10.60 | 1.22 | 0.00 | 1.04 | 0.60 | 0.01 | 3.66 | 0.61 | 0.00 |
| Harpacticoida | 20.00 | 0.98 | 0.00 | 2.08 | 0.40 | 0.00 | 7.32 | 4.06 | 0.01 |
| *Euterpina acutifrons* | 10.60 | 0.56 | 0.00 | - | - | - | - | - | - |
| Tisbidae (n.i.) | 6.62 | 0.17 | 0.00 | - | - | - | - | - | - |
| Longipediidae (n.i.) | 5.96 | 0.23 | 0.00 | - | - | - | 6.10 | 2.03 | 0.01 |
| Harpacticoidea (n.i.) | 1.32 | 0.02 | 0.00 | 2.08 | 0.40 | 0.00 | 1.22 | 2.03 | 0.00 |
| Cyclopoida | 27.50 | 4.63 | 0.02 | 2.08 | 1.00 | 0.00 | 3.66 | 1.01 | 0.00 |
| *Oithona hebes* | 4.64 | 0.26 | 0.00 | - | - | - | - | - | - |
| *Oithona plumifera* | 0.66 | 0.01 | 0.00 | - | - | - | - | - | - |
| *Oncea spp.* | 1.32 | 0.07 | 0.00 | - | - | - | - | - | - |
| *Hemicyclops thalasssus* | 23.18 | 4.01 | 0.02 | 1.04 | 0.08 | 0.00 | 3.66 | 1.01 | 0.00 |
| Cyclopoida (n.i.) | 3.31 | 0.28 | 0.00 | 2.08 | 0.02 | 0.00 | - | - | - |
| Siphonostomatoida (n.i. Caligidae) | 0.66 | 0.01 | 0.00 | - | - | - | - | - | - |
| Copepoda (n.i.) | 15.89 | 1.19 | 0.00 | 8.33 | 2.00 | 0.00 | 13.41 | 4.87 | 0.03 |
